# Concentration and Separation of Active Proteins from Potato Industry Waste Based on Low-Temperature Evaporation and Ethanol Precipitation

**DOI:** 10.1155/2017/5120947

**Published:** 2017-02-19

**Authors:** Sanna Taskila, Mikko Ahokas, Juho Järvinen, Juho Toivanen, Juha P. Tanskanen

**Affiliations:** Faculty of Technology, Chemical Process Engineering Unit, University of Oulu, P.O. Box 4200, 90014 Oulu, Finland

## Abstract

*Purpose*. Potato fruit juice, a residue of starch industry, contains up to 2.5% [w/w] of proteins that are potentially valuable raw-materials of food, cosmetic, and pharma industries. The recovery of protein from the potato fruit juice is limited by the lack of industrially feasible concentration and separation technologies. The present research thus aimed at development of such process for the separation of active protease inhibitors from potato fruit juice.* Methods*. Low temperature mechanical vapor recompression evaporation was applied for concentration of potato fruit juice followed by ethanol precipitation for recovery of active proteins. The effects of precipitation temperature and precipitative agents were investigated employing response surface modeling methodology.* Results*. Concentration of potato fruit juice by evaporation was successful without loss of trypsin inhibition activity. Precipitation using 6.5 M ethanol at low temperature (0–+4°C) was found suitable for the recovery of active protease inhibitors from the concentrate. Piloting at starch industry yielded 50% of total proteins, with a high quantity of active protease inhibitors and a minor inclusion of other proteins.* Conclusion*. Concentration by low-temperature evaporation, followed by ethanol precipitation of protease inhibitors at optimized temperature, is an attractive option for valorization of potato fruit juice.

## 1. Introduction

Potato is a worldwide cultivated agricultural product, with global annual production of over 350 million tons. A remarkable quantity of those potatoes is used in starch industry leading to formation of potato fruit juice (PFJ) as a side stream of starch extraction [1, 2]. The high waste treatment costs have led to an active search of alternatives that would allow value creation from PFJ. Particularly potato proteins, accounting to approximately 2.5% (v/v) of PFJ, are of special interest. Depending on the plant variety and growing conditions, potato contains ca. 10 g/kg of proteins. In case of starch industry, the majority of those proteins end up into the PFJ.

The protein fractions of potato are considered to be nutritionally comparable to that of a whole egg, contributing to the high nutritional potential of the PFJ as well [3]. The main types of potato proteins are patatin, protease inhibitors (PIs), and other higher molecular weight proteins with sizes of 39–45, 4–25, and 30–40 kDa, respectively [3–5].

PIs, representing approximately 50% of potato proteins, have inhibitory activity against serine proteases, cysteine proteases, aspartate proteases, and metalloproteases [6]. The most abundant PIs of potato, denoted as potato serine protease inhibitor, that is, PSPI, possess high inhibitory activity on trypsin and chymotrypsin. PIs have been suggested to affect appetite control system in humans [7]. They are thus mainly considered useful as antinutritional agents. Potato PIs have also proven effects on skin diseases [8], and they may have the ability to lower blood pressure [9, 10].

The separation of proteins from PFJ is traditionally conducted under severe conditions, such as high temperatures or acidity, leading to coagulation and denaturation of proteins. Although coagulation is an effective means to remove proteins from dilute solution, it may also induce irreversible structural changes in the protein molecules, leading to loss of enzymatic activity. Milder technologies are thus needed to allow high-yield recovery of active potato proteins.

Precipitation has been shown to be applicable for the separation of active proteins from PFJ in several earlier studies [5, 11, 12]. The selection of precipitation agent affects largely the yield of active potato proteins. (NH_4_)_2_SO_4_ precipitation may be the most effective means to purify patatin while keeping its lipid acyl hydrolase activity [12]. Ferric chloride (FeCl_3_) has been effective for the isolation of larger than 15 kDa sized protease inhibitors [12]. Metal salts, such as FeCl_3_, act as precipitants disrupting the hydration barriers around the protein molecules. Without the hydration barrier the proteins interact with each other easier causing the formation of aggregates, which will then precipitate from the solution. FeCl_3_ also interacts with the polyphenols. Its role, however, is more of a competitive one since it forms complexes with proteins and also with the polyphenols leaving less polyphenols to interact with proteins [5].

Ethanol is also often used as a precipitation agent as it is miscible with water and thus a poor solvent for proteins. As a nonsolvent, it also has a lower dielectric point than water, increasing the protein-protein attraction forces. Ethanol is also able to form a solution with water, leaving less water to interact with the protein. This is similar to the mechanics involved in the salting-out method. As a commonly used precipitation agent ethanol is known to remain the activity of proteins, which means that it is safe to use with proteins products with conformity.

The present precipitation processes are also limited by the low concentration of proteins in PFJ, namely, 1–3% [1]. The recovery of active proteins without preliminary concentration is thus rather resource-efficient with high chemical consumption and large processing volumes. The objective of present research was thus to develop an energy and material efficient process that would be applicable for the recovery of active PIs from dilute PFJ produced in starch industry. The scope was on scalable technologies that allow high yield of active proteins without excessive chemical or energy consumption and high processing capacity and avoid need for extensive down-stream processing. For this purpose, a procedure involving MVR evaporation at mild conditions for the concentration of PFJ, followed by ethanol precipitation of protein at low temperature, is proposed.

## 2. Material and Methods

### 2.1. PFJ Concentrates

PFJ samples for initial precipitation experiments were prepared from commercial potatoes (cultivar Challenger) as described in previous studies [5]. Briefly, peeled potatoes were first ground in water with a domestic appliance grade blender, after which the potato solids were sieved out ending up with the PFJ. Industrial PFJ samples from a cooperating starch producer were also used for the confirmation of the viability of the concentration and recovery technology.

The in-house produced PFJ was concentrated by evaporation at 40°C and 35 mbar for removal of excess water (laboratory scale equipment, Büchi Rotavapor R-153, and Büchi Vacuum System B-178). The industrial PFJ samples were concentrated in situ using pilot-scale single-state falling film mechanical vapor recompression (MVR) evaporation equipment (Epcon Evaporation Technology AS, Norway). The processing capacity of pilot-scale evaporation was 100 L/h of PFJ. In MVR evaporation technology the vapor is compressed in a fan or a compressor to a higher temperature and pressure and used as energy source instead of boiler steam, leading to reasonably lower energy consumption for the water purification process (based on our experience 10–25 kWh/m^3^).

### 2.2. Design of Experiments and Response Surface Modeling

Response surface methodology (RSM) using MODDE software for Design of Experiments and Optimization (Version 8.0.0.0, UMETRICS AB, Umeå, Sweden) was applied for the modeling of factor effects on recovered activity of PIs. The RSM was prepared using quadratic face centered central composite design (CCF). The performances of the models were evaluated by assessing analysis of variance (ANOVA) as described in [13]. Briefly, with good models, the standard deviation of the model should be much larger than the standard deviation of the noise with its upper confidence level. The effect of a factor can be considered statistically significant if the confidence interval is not larger than the effect itself. Factors under investigation were ethanol concentration (0–6.5 M), FeCl_3_ concentration (0–25 mM), and temperature (0–20°C). The experiment matrix is presented in Table 1. The iteration algorithm for optimization was Nelder-Mead method [14].

### 2.3. Precipitation

The initial development of precipitation process was carried out in total volume of 3 mL. Precipitation agents and 170 g/l of PFJ were added to each reaction according to Table 1. With the ferric chloride EDTA was added to the resolubilization buffer to inhibit the effect of the metal ions' charge on the dissolving of the proteins to the liquid [5]. Reactions were incubated under agitation in Thermomixer Comfort (Eppendorf, Germany) for 1 h. After precipitation the reactions were centrifuged at 11000 ×g and +4°C to recover precipitated proteins. Active proteins were recovered by dissolving to 100 mM sodium phosphate buffer (pH 7.0) and incubation at 20°C and 400 rpm for 1 h. Nonsoluble fraction was removed by centrifugation. The precipitation experiments of PFJ concentrates from starch industry were carried out by addition of 1 L of 96% ethanol to 1 L of PFJ concentrate (48% ethanol) at +4°C.

### 2.4. Protein Quantity and Quality Analyses

Sodium dodecyl sulphate polyacrylamide gel electrophoresis (SDS-PAGE) was used for the control of produced protein fractions. PageRuler™ Plus Prestained Protein Ladder (Thermo Scientific) was used as marker in SDS-PAGE. The total protein content was measured by means of standard BCA assay (The Thermo Scientific Pierce BCA Protein Assay Kit).

### 2.5. Protease Inhibitor Activity Measurement

The solubility of potato proteins correlates to the structure of potato proteins; that is, irreversible structural changes induced during the precipitation show poor resolubility of the protein from the precipitate. Thus the resoluble protein yield can be used as a measure for recovery of active proteins [15]. Moreover, the resoluble protein yield can be quantitatively determined. Therefore, the quantity of the resoluble proteins was used for the evaluation of active protein yields. However, to verify that the activity of PIs is remained during processing, trypsin inhibition assay was prepared. Trypsin disintegrates methyl ester of N-*α*-tosyl-L-arginine (TAME, extinction coefficient 449.5 l/mol*∗*cm). Disintegration can be detected with spectrophotometer at 247 nm (UV). In order to determine trypsin inhibition activity in the isolated protein fractions, the samples were first diluted to reach total protein concentration of approximately 2–4 g/l.

**Figure 1:**
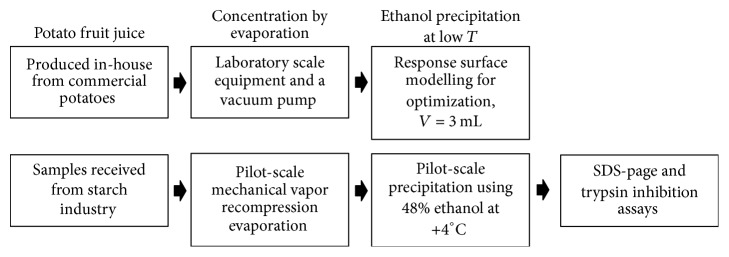
Experimental procedures in the present research. The upper row represents the procedure to optimize the precipitation conditions using commercial potatoes. The lower row represents the procedure to utilize optimized conditions at starch industry for the pilot-scale precipitation.

The experimental procedures are summarized in Figure 1.

## 3. Results

### 3.1. Effect of Precipitation Agents and Temperature on Protein Recovery from PFJ

**Figure 2:**
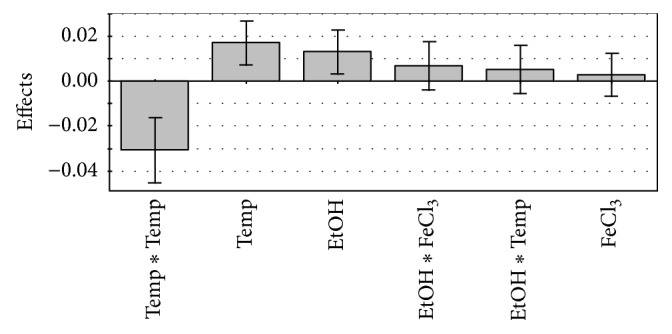
The effects of precipitation agents and temperature on recovery of resoluble proteins from potato. The scaled effects of the model terms are presented at 95% confidence level. Temp: temperature, EtOH: ethanol concentration, FeCl_3_: FeCl_3_ concentration, Temp *∗* Temp: square term of temperature, EtOH *∗* FeCl_3_: interaction term of ethanol and FeCl_3_, and EtOH *∗* Temp: interaction term of ethanol and temperature.

Effects of ethanol, FeCl_3_, and temperature on yield of resoluble potato PIs from laboratory prepared PFJ were investigated by means of RSM. Resoluble protein concentrations recovered in precipitation are presented in Table 2. To improve fit of model response transformation was used as described in [13]. According to* F*-test, levels of ethanol and temperature affected yield of resoluble potato proteins (*p* < 0.05, Figure 2). The effect of FeCl_3_ was not statistically significant.

**Figure 3:**
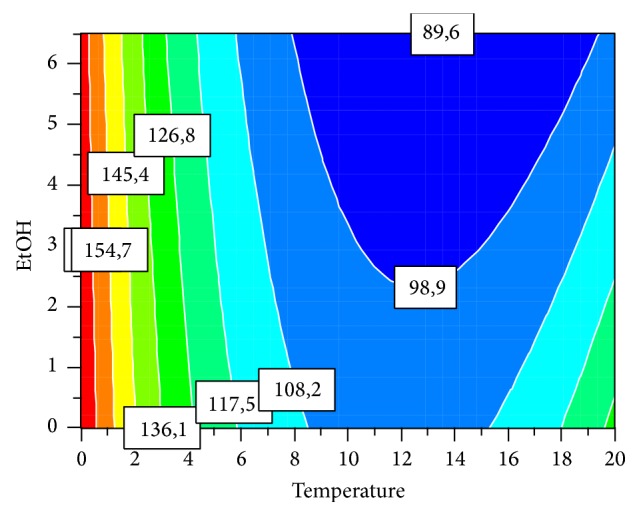
Prediction of favorable ethanol concentration and temperature within the investigated region to achieve highest possible quantity of resoluble proteins.

The Nelder-Mead simplex optimization within the studied region suggested use of 6.5 M ethanol and temperature of 0°C for maximal recovery of proteins (Figure 3).

### 3.2. Recovery Efficiency of Proteins from Starch Industry PFJ

The efficiency of the precipitation for recovery of active proteins from industrial PFJ was investigated using concentrate from the pilot evaporation. The precipitation was carried out at ethanol concentration of 10.4 M and temperature of +4°C due to practical reasons at the industrial site.

**Figure 4:**
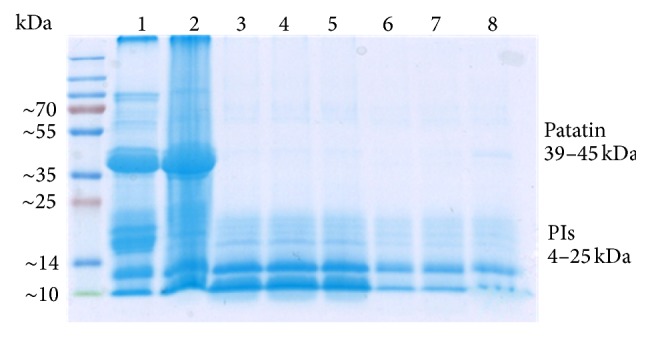
SDS-PAGE of proteins in native PFJ and its processed fractions. 1: PFJ; 2: concentrate; 3, 4, and 5: supernatant from precipitation with 10.4 M ethanol (in triplicate); 6, 7, and 8: resolubilized protein in Tris buffer (in triplicate).

It can be observed in the SDS-PAGE (Figure 4) that both main protein fractions, PIs and patatin, were present in soluble form in the original PFJ feedstock. Evaporation did not seem to affect the quality of patatin (39–45 kDa) or low molecular weight PIs (4–25 kDa), but some decrease in the band intensity is seen between 14 and 25 kDa. During the ethanol precipitation, the majority of patatin was precipitated irreversibly as only a minor quantity of patatin is present in the resolubilized fraction. PIs were divided into ethanol and precipitate and the majority of PIs could be recovered in ethanol or resolubilized from the precipitate.

According to the mass balance the total protein concentration in the original PFJ was 13 g/l from which approximately 38% was left nonprecipitated in the ethanol phase (Table 3). The rest of the proteins were precipitated. Approximately 12% of the total protein was recovered by resolubilization from the precipitate (Figure 4 and Table 4). The activity of resoluble PIs was confirmed by measurement of the trypsin inhibition activity (Table 4). The relative inhibition activity was calculated by dividing the activity by the quantity of total protein in the sample.

The trypsin inhibition activity per gram of protein was highest in the nonprecipitated fraction that remained after incubation in ethanol (Table 4). In this fraction patatin was present only in minor quantity. Some active PIs could also be recovered after precipitation by resolubilization. Altogether, the soluble fractions represent protein yield of 50% from total protein in PFJ, containing a major portion of active PIs and a minor fraction of patatin. The concentration of PFJ by means of low-temperature evaporation had a minor effect on trypsin inhibition activity.

## 4. Discussion

The present research focused on development of an industrially feasible and energy-efficient process to recover active proteins from starch industry residues. At the beginning of this study the combination of ethanol and FeCl_3_ for precipitation was considered as the most feasible option for the purpose. It was, however, noticed that the use of FeCl_3_ did not improve the yield of soluble proteins at the examined conditions which may relate to conformational changes of PIs induced by FeCl_3_ [12]. Another disadvantage of FeCl_3_ and other salts is the need for their removal from produced protein fractions.

Based on the present results and previously reported investigations, ethanol precipitation allows satisfactory recovery of active PIs from PFJ [12]. Since ethanol can be recovered from the protein concentrate by means of low-temperature evaporation, principally using the same methodology compared to that in the concentration of PFJ, and circulated back to the precipitation process, we have considered it as the most feasible precipitation agent for the recovery of proteins in starch industry.

Regarding targeted purification of PIs, ethanol seems well suitable for maintaining high chymotrypsin activity while other agents may be considered for higher yield of trypsin inhibiting fraction of PIs [12]. However, to maintain overall feasibility of the procedure, we suggest that the use of other precipitation agents is considered only in case that their purification from the produced protein fractions can be arranged in a cost-efficient manner which reflects the benefit from improved active PI yield.

The yield of resoluble, potentially active, proteins was shown to depend on both ethanol concentration and precipitation temperature of which result is also in line with previous reports [11, 12]. Based on iterative optimization even higher ethanol concentrations and lower temperatures may be used than those actually investigated in the experiments. It was thus concluded that excess ethanol may be used in the pilot experiments to ensure sufficient precipitation and that the temperature should be as low as possible for efficient recovery. However, in the further process development it might be advisable to optimize the conditions for the precipitation with respect to the technoeconomic performance of the process.

The concentration of PFJ by means of low-temperature evaporation allowed a nearly 6-fold concentration of potato proteins while only a minor decrease in the relative trypsin inhibition activity was detected. The proposed MVR evaporation technology is thus considered a feasible choice for the further process development. In the future investigations the overall feasibility evaluation of the concentration and precipitation process will be constructed.

## 5. Conclusion

Low-temperature evaporation and ethanol precipitation were successfully used for the recovery of active PIs from industrial PFJ. The evaporation, precipitation, and resolubilization procedure remained the trypsin inhibition activity of the PIs. The proposed process can be developed for an industrially feasible means for the treatment of starch industry waste waters.

## Figures and Tables

**Table 1:** Experiment matrix for RSM.

Exp number	Exp name	EtOH [M]	FeCl_3_ [mM]	*T* [°C]
1	N1	0	0	0
2	N2	6.5	0	20
3	N3	0	0	20
4	N4	0	25	0
5	N5	3.25	0	10
6	N6	3.25	12.5	10
7	N7	3.25	25	10
8	N8	3.25	25	20
9	N9	0	25	20
10	N10	6.5	12.5	10
11	N11	6.5	25	0
12	N12	0	12.5	10
13	N13	6.5	0	0
14	N14	3.25	12.5	10
15	N15	6.5	25	20
16	N16	3.25	12.5	0
17	N17	3.25	12.5	20
18	N18	3.25	12.5	10

**Table 2:** Resoluble protein concentration in precipitates.

Exp number	Variable levels [EtOH/FeCl_3_/*T*]	Protein [g/L]
1	0/0/0	14.25
2	6.5/0/20	5.80
3	0/0/20	37.47
4	0/25/0	29.76
5	3.25/0/10	24.13
6	3.25/12.5/10	10.08
7	3.25/25/10	17.35
8	3.25/25/20	13.25
9	0/25/20	29.08
10	6.5/12.5/10	25.21
11	6.5/25/0	8.75
12	0/12.5/10	22.31
13	6.5/0/0	10.35
14	3.25/12.5/10	13.11
15	6.5/25/20	16.89
16	3.25/12.5/0	16.32
17	3.25/12.5/20	16.94
18	3.25/12.5/10	19.76

**Table 3:** Mass balance of total protein in the PFJ and its processed fractions.

Fraction	Protein	Protein
	g/l	g/kg of PFJ
PFJ	13	13
Concentrate	74	13
Nonprecipitated (soluble)	19.6	5
Precipitated (resoluble)	18.5	1.5
*Total soluble protein*		*6.5*

**Table 4:** Trypsin inhibition activity of native PFJ and processed concentrates.

Fraction	*x* dil.	Reaction	Trypsin	Relative
		rate	inhibition	inhibition
		abs/min	%	/g protein
Reference	—	0.075	0	—
PFJ	10	0.007	91	9.3
Concentrate	60	0.016	79	8.5
Nonprecipitated (soluble)	8	0.012	84	11.9
Precipitated (resoluble)	20	0.008	89	5.4
